# Microgravity crystallization of perdeuterated tryptophan synthase for neutron diffraction

**DOI:** 10.1038/s41526-022-00199-3

**Published:** 2022-05-04

**Authors:** Victoria N. Drago, Juliette M. Devos, Matthew P. Blakeley, V. Trevor Forsyth, Andrey Y. Kovalevsky, Constance A. Schall, Timothy C. Mueser

**Affiliations:** 1 Department of Chemistry and Biochemistry, University of Toledo, Toledo, OH 43606 USA; 2 Life Sciences Group, Institut Laue-Langevin, 38000 Grenoble, France; 3 Partnership for Structural Biology (PSB), 38000 Grenoble, France; 4 Large-Scale Structures Group, Institut Laue-Langevin, 38000 Grenoble, France; 5 Faculty of Natural Sciences, Keele University, Staffordshire, ST5 5BG UK; 6 Faculty of Medicine, Lund University, and LINXS Institute for Advanced Neutron and X-ray Science, Lund, Sweden; 7 Neutron Scattering Division, Oak Ridge National Laboratory, Oak Ridge, TN 37830 USA; 8 Department of Chemical Engineering, University of Toledo, Toledo, OH 43606 USA

**Keywords:** Structural biology, Enzyme mechanisms

## Abstract

Biologically active vitamin B_6_-derivative pyridoxal 5′-phosphate (PLP) is an essential cofactor in amino acid metabolic pathways. PLP-dependent enzymes catalyze a multitude of chemical reactions but, how reaction diversity of PLP-dependent enzymes is achieved is still not well understood. Such comprehension requires atomic-level structural studies of PLP-dependent enzymes. Neutron diffraction affords the ability to directly observe hydrogen positions and therefore assign protonation states to the PLP cofactor and key active site residues. The low fluxes of neutron beamlines require large crystals (≥0.5 mm^3^). Tryptophan synthase (TS), a Fold Type II PLP-dependent enzyme, crystallizes in unit gravity with inclusions and high mosaicity, resulting in poor diffraction. Microgravity offers the opportunity to grow large, well-ordered crystals by reducing gravity-driven convection currents that impede crystal growth. We developed the Toledo Crystallization Box (TCB), a membrane-barrier capillary-dialysis device, to grow neutron diffraction-quality crystals of perdeuterated TS in microgravity. Here, we present the design of the TCB and its implementation on Center for Advancement of Science in Space (CASIS) supported International Space Station (ISS) Missions Protein Crystal Growth (PCG)-8 and PCG-15. The TCB demonstrated the ability to improve X-ray diffraction and mosaicity on PCG-8. In comparison to ground control crystals of the same size, microgravity-grown crystals from PCG-15 produced higher quality neutron diffraction data. Neutron diffraction data to a resolution of 2.1 Å has been collected using microgravity-grown perdeuterated TS crystals from PCG-15.

## Introduction

Pyridoxal 5′-phosphate (PLP) dependent enzymes are a functionally diverse family of enzymes with seven identified Fold Types that are responsible for over 140 different distinct enzymatic activities^1,2^. Catalytic versatility is credited to the organic cofactor pyridoxal, the biologically active derivative of pyridoxamine, vitamin B_6._ The cofactor facilitates a broad range of chemistry including transamination, racemization, phosphorylation, α-decarboxylation, aldol cleavage, β- and γ-elimination and replacement reactions, and glycogen phosphorylation^3^. PLP-dependent enzymes are located primarily in the amino acid metabolic pathways, including the interconversion of α-amino acids and the biosynthesis of antibiotic compounds^4,5^. PLP-dependent enzymes are attractive targets for specific inhibitor design with pertinent examples such as DOPA decarboxylase, which has been implicated in treatment of Parkinson’s Disease, and alanine racemase, an important enzyme in bacterial cell wall synthesis^6^.

**Fig. 1:**
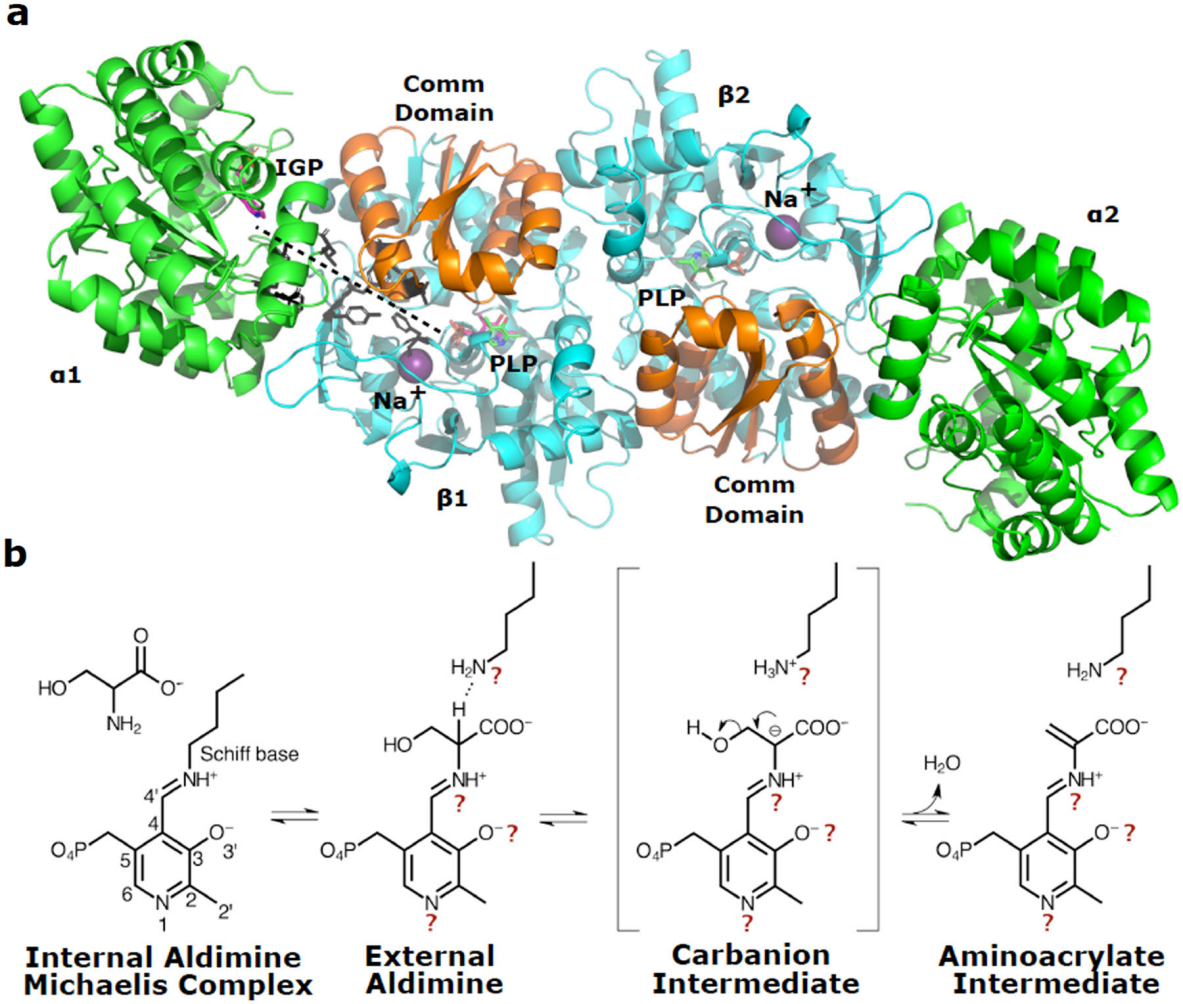
*Salmonella typhimurium* tryptophan synthase structure and reaction mechanism. **a** The TS αββα tetramer ribbon model (α in green and β in cyan and orange) with indole-3-glycerol (IGP) phosphate bound in the α-active site and pyridoxal 5′-phosphate bound as the internal aldimine in the β-active site (PDB 1BKS and 1QOQ). The cleavage of IGP in the α-reaction releases indole, which travels down a 25 Å hydrophobic channel (dashed line, residues lining the channel shown in black) to the β-active site and couples to a PLP-activated serine (aminoacrylate). The monovalent cation site (occupied by Na^+^ shown as a purple sphere) is involved in coordinating the two reactions. Motion in the communication (Comm) domain (orange) of the β-subunit defines the open and closed conformations of the enzyme. **b** PLP activation of L-Ser in the TS β-active site initiates with the binding of L-Ser as a Michaelis complex that is likely coupled to the presence of indole in the hydrophobic channel. Numbering of the PLP ring is depicted in the internal aldimine Schiff base structure. The unprotonated α-amino group of L-Ser links to the internal aldimine protonated Schiff base replacing the catalytic lysine (Lys 87) to form the external aldimine. Lys 87 then acts as a general base to abstract a proton from the substrate Cα to form the transient carbanionic intermediate, which proceeds through β-elimination to release water and form the reactive aminoacrylate intermediate. Question marks denote atoms where the protonation state is of particular interest. Question marks were omitted from the internal aldimine structure for simplicity.

Here, we discuss the Fold Type II PLP-dependent enzyme tryptophan synthase, an essential enzyme from the human enteric pathogen, *Salmonella typhimurium* (Fig. 1a). The αββα hetero-tetramer of TS is responsible for catalyzing the final steps in the biosynthesis of L-tryptophan, linking serine activated by β-elimination with indole in a replacement reaction. In the α-subunit, a retro-aldol cleavage of indole 3-glycerol-phosphate produces indole and d-glyceraldehyde-3-phosphate. PLP is located in the β-subunit active site, coupled to Lys 87 in a Schiff base internal aldimine state. The L-Ser substrate displaces the active site lysine through a gem-diamine intermediate to form a Schiff base external aldimine. The cofactor promotes β-elimination of the substrate releasing the L-Ser hydroxyl group, resulting in the formation of the highly reactive aminoacrylate intermediate (Fig. 1b). Indole travels through a 25 Å hydrophobic channel connecting the α- and β-subunit active sites and couples in a replacement reaction to the aminoacrylate, producing the final product, L-Trp^7–9^.

L-Trp is a dietary essential amino acid in humans. With no human homolog, TS is an attractive target for inhibition of pathogenic organisms, such as *Salmonella enterica* and *Staphylococcus aureus*. TS has also become the focus of many directed-evolution studies, including the work, which earned the 2018 Nobel Prize in Chemistry, for the synthesis of non-canonical amino acids and tryptamine products for use in pharmaceutical developments^10^. Catalytic diversity of PLP-dependent enzymes are unified by the common step of converting the cofactor from the resting internal aldimine state (Fig. 1b) to the active substrate-bound external aldimine. Divergence in reaction specificity occurs from this point in the reaction mechanism^11^. Hydrogens are shuffled around during the complex enzymatic reaction catalyzed by TS. Knowledge of where the hydrogens are located and where they move to is indispensable in understanding TS specificity and in aiding the structure-based design of novel antibiotics. How such specificity is achieved is still not well understood. This level of comprehension can only be achieved through detailed atomic-level structural studies of reaction intermediates^1^. Structure-based drug design primarily utilizes the three-dimensional models obtained from single-crystal X-ray diffraction. These models are incomplete since hydrogen atoms, which constitute ~50% of the atoms in a protein, are not visible^1^. The ability to accurately and directly determine the positions of hydrogen atoms and hence assign protonation states to key amino acid side chains and the PLP cofactor is essential.

Single-crystal neutron diffraction is a powerful technique for directly visualizing the positions of hydrogen atoms. The isotopic exchange of hydrogen atoms in macromolecules by deuterium atoms results in a significant increase in neutron scattering power that renders hydrogen atoms (as deuterium) as visible as the carbon and nitrogen atoms. In addition, perdeuteration (i.e., all H replaced by D) results in the elimination of the large incoherent scattering from hydrogen thereby vastly improving the signal-to-noise ratio of the data and decreasing the crystal volumes needed for a successful neutron crystallographic analysis. Despite this, the low fluxes of neutron sources typically require crystal volumes of ~0.5 mm^3^ to provide reasonable diffraction, although in some cases crystals can be smaller^12–14^. This bottleneck can be extremely challenging to overcome as large protein crystals often develop defects that affect diffraction quality. Most crystal defects are a result of gravity-driven convection currents present in solution, which result in irregular crystal feeding and depletion zones^15,16^. Microgravity offers a unique environment to improve macromolecular crystal growth through elimination of major convection sources.

Microgravity crystallization efforts began in the 1980s and successful missions justified the development of specialized hardware to facilitate long-term crystal growth aboard the International Space Station (ISS). Flight hardware were initially designed for rapid equilibration on shuttle missions, aimed at producing diffraction-quality crystals for X-ray crystallography. Over the years, several different apparatus designs have flown to the ISS. Some of the more commonly flown apparatuses are the advanced protein crystallization facility (APCF), vapor diffusion device (VD), protein crystallization facility (PCF), vapor diffusion apparatus (VDA), protein crystallization apparatus for microgravity (PCAM), and diffusion-controlled crystallization apparatus for microgravity (DCAM)^17,18^. Crystals with improved quality and X-ray diffraction resolution were obtained but these devices were not designed to produce the excessively large size of crystals necessary for neutron crystallographic studies. An increased enthusiasm in the structural biology community towards neutrons is due to the availability of improved neutron beamlines throughout the world, such as LADI-III, Institut Laue-Langevin, Grenoble, France, and MaNDI, Spallation Neutron Source, Oak Ridge National Laboratory, USA^19–21^. A priority in recent years has been to develop new devices designed specifically to utilize microgravity for neutron-quality crystal growth.

The flight hardware, Granada Crystallization Box (GCB), relies on gel-barrier capillary diffusion and has successfully produced very large crystals for neutron diffraction studies^22^. Here, we report similar hardware, the TCB, which relies on membrane-barrier capillary-dialysis microgravity crystal growth. The implementation of the TCB for the crystal growth of perdeuterated TS on the ISS for neutron diffraction studies is discussed. The design allows for modulation of equilibration times and the ability to sample a range of crystallization conditions while being highly cost effective. TS crystallizes rapidly and the plate-like crystals develop severe inclusions that result in limited size, high mosaicity, and poor diffraction quality. Mosaicity is an indicator of crystal quality, measuring the spread of microdomain orientations within a crystal. Reducing mosaicity increases signal-to-noise^23^. As we have not been able to produce TS crystals of neutron diffraction quality in unit gravity, we turned to microgravity crystallization aboard the ISS to facilitate the growth of larger crystals with reduced mosaicity. We succeeded in growing TS crystals that diffract neutrons to 2.1 Å resolution and demonstrated that the crystals grown in microgravity on the ISS give superior neutron diffraction compared to controls grown in unit gravity in the TCBs.

## Methods

### Perdeuteration of TS

Perdeuterated tryptophan synthase (TS) protein from *Salmonella Typhimurium* was produced in the Deuteration Laboratory in the Life Sciences Group of the Institut Laue-Langevin (ILL, Grenoble, France)^24^. To obtain perdeuterated Tryptophan Synthase protein, a transposition reaction was first carried out on the original pEBA-10 vector in order to modify the resistance selection marker from ampicillin to kanamycin. The Tn5 transposon insertion kit (EZ-Tn5TM <KAN-2> Insertion Kit) from Epicentre® Biotechnologies (an illumina® company, available through Lucigen®) was used for this transposition reaction. New kanamycin-resistant pEBA-10 plasmids containing cDNA coding for both the α- and β- subunits of TS were transformed into One ShotTMBL21(DE3) *E. coli* cells (Invitrogen). Cells were adapted to deuterated Enfors minimal medium, containing d8-glycerol (99% deuterium; Eurisotop) as carbon source and D_2_O as solvent, in the presence of kanamycin at a final concentration of 35 μg/mL^24^. Three rounds of adaptation were carried out, each of them overnight, by diluting cells in fresh deuterated medium and regrown, before protein expression was investigated. For production of pD-TS, deuterated Enfors medium was supplemented in a 1:1 (v/v) ratio with ^2^H-labeled Rich growth *E. coli* media OD2 (Silantes) keeping kanamycin at a final concentration of 35 μg/mL. A total of eighteen 2 L flasks with baffles were used, each containing about 340 mL of the fully deuterated growth medium mixture. The bacteria were grown at 37 °C to an OD_600_ of 0.7–0.8, the temperature was then decreased to 30 °C and protein expression was induced by addition of isopropyl β-d-1-thiogalactopyranoside (IPTG) to a final concentration of 1 mM. After 15 h of induction at 30 °C and 150 rpm, cells were harvested by centrifugation. Cultures yielded a total of 38 g of cell paste with very good expression levels of both subunits of TS. Perdeuterated TS produced was purified and crystallized using H_2_O-based buffers following the standard protocols developed for the H-TS protein.

### Purification of TS

H- and pD-TS were purified as previously described with slight modification^25^. Cell pastes were resuspended in 50 mM Tris-HCl pH 7.8, 1 mM EDTA, 10 mM β- mercaptoethanol (β-Me), and 0.2 mM pyridoxal 5′-phopshate (PLP) at 5 mL/g of cell paste. Phenylmethane sulfonyl fluoride (PMSF), polyethyleneimine (PEI), and lysozyme were added to the suspension in the concentrations of 1 mM, 0.2% (w/v), and 0.1 mg/mL, respectively. The suspension was stirred on ice for 30 min and sonicated with Branson Sonifer 250. The lysates were centrifuged to clarity at 12,000 rpm at 4 °C. The soluble fraction was brought to 6% PEG 8000 and 5 mM spermine and centrifuged at 12,000 rpm (4 °C) for 10 min. The soluble fraction was brought to 12% PEG 8000 and stored at 4 °C for 24–48 h. The mixture was centrifuged at 12,000 rpm (4 °C) for 30 min to collect precipitated TS microcrystals. The crystals were washed with 12% PEG and resuspended in 50 mM Bicine pH 7.8, 1 mM EDTA, 10 mM β-Me, and 0.2 mM PLP. The sample was dialyzed against the buffer overnight. Ammonium sulfate was added to the soluble sample dropwise to 60% and stirred on ice for 30 min. Precipitant was collected by centrifugation at 12,000 rpm (4 °C) for 45 min. The precipitant was resuspended in 50 mM Bicine pH 7.8, 1 mM EDTA, 10 mM β-Me, and 0.2 mM PLP. The sample was further purified using a Superdex-75 sizing column (1 × 30 cm) and verified with SDS-PAGE. The homogeneity of TS samples used in crystallization were confirmed by dynamic light scattering with Anton Paar Litesizer 500.

### Toledo Crystallization Boxes (TCBs)

The TCBs consist of a polypropylene box (Ultra Pro, ASIN, B00AU762PK) containing 50 capillaries sealed in individual polyethylene bags (prepared from 1” LDPE clear poly tubing). Quartz capillaries (VitroCom, CV2024-Q-100) with an inner diameter of 2 mm and a thickness of 0.2 mm were cut to 5 cm in length. 3.5–5 kDa MWCO dialysis tubing (Biotech RC tubing, Spectrum Labs, 133198) was secured to the end of the capillary with a piece of Tygon tubing (Cole Parmer, 0.094” ID, 95802-03) cut to 1 cm with holes made using a 1 mm fabric hole punch. The capillary was filled with protein (150 μL) using a blunt-end needle (Hamilton, 22 Ga. 90134) and 1 mL disposable syringe. Beeswax (Hampton Research, HR4-312) was melted, and the end of the capillary sealed using multiple rapid-dipping with air cooling between steps to minimize sample heating. The sealed capillary and 3 mL of mother liquor were placed in a polyethylene bag and heat sealed. Protein and solutions were degassed prior to assembly of the TCB. The equilibration times of TCBs with various MWCO dialysis membranes was determined for PEG 8000. A standard curve was constructed using water in place of protein in the capillary. The refractive index of the solution in the capillary was taken in triplicate between 5 and 315 h on a Milton Roy Abbe Refractometer. The equilibration rates were determined for both the horizontal and vertical positions.

### Protein Crystal Growth (PCG)-8

Crystallization mission PCG-8 was aboard SpaceX’s CRS-15 that launched from Space Launch Complex 40 at Cape Canaveral Air Force Station (CCAFS) in Florida on Friday, June 29, 2019 at 5:42 a.m. EDT. The Dragon capsule docked on the ISS on July 2, 2018 at 10:54 UTC. European Space Agency astronaut Alexander Gerst, part of ISS Expedition 56, transferred the TCB devices to storage. TCB devices containing TS samples were stored at ISS in an ambient storage locker at 22 °C. Equivalent ground control experiments were stored at 22 °C in a Fisher Scientific Low Temperature Incubator (Model # FFU20F9FW0) in the vertical position. The TCB devices returned to Earth in the Dragon capsule that splashed down in the Pacific Ocean on August 3, 2018 at 6:17 p.m. EDT.

### Protein Crystal Growth (PCG)-15

Crystallization mission PCG-15 was aboard SpaceX’s CRS-18 that launched from Space Launch Complex 40 at Cape Canaveral Air Force Station (CCAFS) in Florida on Thursday, July 25, 2019 at 6:01 p.m. EDT. The Dragon capsule docked on the ISS on July 27, 2019 at 13:11 UTC. NASA astronauts Christina Koch and Nicklaus Hague, part of ISS Expedition 60, transferred the TCB devices to storage. TCB devices containing H-TS and pD-TS samples were stored at ambient temperature (22 °C) both on the ISS and in unit gravity in the same manner as PCG-8. Capillaries were filled with 20 mg/mL protein solution. Crystallization conditions of pD-TS were 50 mM Bicine pH 7.8, 1 mM EDTA, 0.2 mM PLP, 2 mM spermine, and 6–8% PEG 8000. A small population of capillaries from each percentage of PEG sampled also contained 5% ethylene glycol as an additive. The TCB devices remained on the ISS for ~6 months and returned to Earth with SpaceX’s CRS-19 Dragon capsule. The capsule splashed down in the Pacific Ocean on January 7, 2020 at 10:41 a.m. EST.

### X-ray data collection and processing

Room temperature X-ray diffraction data from PCG-8 were collected on IMCA-CAT (17-ID-B) equipped with a Dectris Eiger2 X 9M detector at the Advanced Photon Source (APS) in Chicago. The data were indexed and integrated with Mosflm and scaled using SCALA in the CCP4 Program Suite^26,27^.

Room temperature X-ray diffraction data from PCG-15 were collected on a Rigaku HighFlux HomeLab instrument at Oak Ridge National Laboratory equipped with an Eiger 4M detector. Data were indexed, integrated, and scaled using Rigaku’s CrysAlis Pro software. Phasing was solved by molecular replacement in PHENIX using the PDB entry 1BKS^28^. Manual manipulation of the models was performed with Coot between refinements using phenix.refine^29^.

### Neutron data collection and processing

All crystals used in neutron diffraction experiments were vapor H/D exchanged prior to data collection. The solution used for vapor H/D exchange was equivalent to the condition used in crystallization. Concentrated stock solutions of all reagents were prepared in D_2_O and diluted in D_2_O to prepare the final exchange solution. Crystals were equilibrated against the deuterated exchange solution for a minimum of 2 weeks prior to data collection. Neutron diffraction was tested from the microgravity- and ground-grown crystals of ~0.2 mm^3^ in volume each on IMAGINE macromolecular diffractometer at the High Flux Isotope Reactor (HFIR, Oak Ridge National Laboratory)^30–34^. Diffraction from each crystal was first evaluated by collecting 16-hour images using the Laue mode with the neutron wavelengths in the range of 2.8–10 Å. Both crystals diffracted sufficiently well to allow the diffraction patterns to be indexed using the Daresbury Laboratory LAUE suite program LAUEGEN modified to account for the cylindrical geometry of the detector^35,36^. Quasi-Laue neutron diffraction images with the neutron wavelengths in the range of 2.8–4.5 Å were then collected for each crystal using 24-hour exposures. The quasi-Laue images were indexed using the crystal orientation matrices obtained from the Laue images and the diffraction spots were integrated to 3.2 Å for the microgravity crystal and to 4.0 Å for the ground crystal, demonstrating substantially stronger neutron diffraction from the space crystal.

Neutron quasi-Laue diffraction data from a crystal of microgravity-grown pD-TS were collected at room temperature using the LADI-III diffractometer at the Institut Laue-Langevin^20^. A neutron wavelength range (∆*λ*/*λ*~30 %) of 2.85–3.80 Å was used for data collection with diffraction data extending to 2.1 Å resolution. The crystal was held stationary at different φ (vertical rotation axis) for each exposure. A total of 57 images were recorded from four different crystal orientations. The neutron diffraction images were processed using the *LAUEGEN* software^35,36^. The program *LSCALE* was used to determine the wavelength normalization curve using intensities of symmetry-equivalent reflections measured at different wavelengths. The data were merged and scaled using *SCALA*^27,37^.

## Results and discussion

Two capillary-dialysis microgravity crystal growth experiments for TS were conducted on the International Space Station. A short duration (5-weeks) test experiment (CASIS PCG-8), one TCB containing hydrogenated TS (H-TS), were delivered and returned by SpaceX CRS-15. A long duration (6-months) production crystal growth (CASIS PCG-15) of perdeuterated TS (pD-TS) was delivered on SpaceX CRS-18 and returned on SpaceX CRS-19. Both flights utilized three boxes, each box contained 45 to 50 capillaries and each capillary held ~3 mg of protein. This small, inexpensive approach was capable of subjecting ~0.5 g of protein to microgravity crystallization, yielding a diverse population of diffraction-quality crystals with several having the volume necessary for neutron diffraction.

### Crystallization methods for growth of large (>1 mm^3^) crystals

The advent of synchrotron radiation and micro-focused beams have made the endeavor of growing large crystals nearly obsolete for macromolecular X-ray crystallography. Neutron sources, on the other hand, have comparatively much lower fluxes and thus require larger crystals to compensate for this. The main methods used to grow large protein crystals in unit gravity are vapor diffusion, batch, counter-diffusion, and dialysis^16^.

Vapor diffusion crystallization is the most common crystallization method for proteins. It is performed by mixing protein solution with crystallization condition and equilibrating the drop against a reservoir of the mother liquor. For large drop volumes, sitting-drop vapor diffusion in 9-well glass plates enclosed in a sandwich box is preferred (Hampton Research HR3-136). The batch method involves direct mixing of the protein and mother liquor and can be performed in glass vials or sealed 9-well glass plates. In counter-diffusion, the protein is held within the capillary and the crystallization solution is separated by a permeable barrier, commonly agarose gel. Separating the protein from mother liquor by dialysis membrane, dialysis crystallization is typically performed in dialysis buttons (Hampton HR3-336), which do not have the confined geometry at the diffusion interface that is characteristic of counter-diffusion^38^.

Convection-driven mass transport in vapor diffusion and batch crystallization creates depletion zones that promote defects in crystals. Mass transport by convection in counter-diffusion crystallization is reduced and the mass transport process is driven primarily by diffusion, making this a preferred method for large crystal growth for some proteins^15,16^. Despite lessening the effects of convection in comparison to other methods of crystallization, counter-diffusion still suffers from the influences of gravity. The only way to eliminate mass transport by convection is in the absence of gravity. One method of simulating a gravity-free environment in a unit gravity crystallization experiment is to grow crystals in a gel, typically agarose^39^. This involves diffusion of mother liquor into a gelation of low-melting point agarose and protein. Crystals grown in the gel must be removed from the diffusive media prior to neutron data collection as agarose will contribute to a higher background. Removing crystals requires dissecting them away from the gel and risks damage incurred by manual manipulation. Even remnants of gel adhered to the surface of crystals during data collection may reduce the signal-to-noise by increasing the incoherent scattering. In addition, making a gel of the protein solution imposes the risk of heat damage to the protein. While in-gel crystallization is more accessible than the microgravity environment of the ISS, its use in growing crystals for neutron diffraction has been limited.

Early microgravity crystallization experiments were enabled by the prosperity of the Space Shuttle program. The resurgence of space flight and motivation to utilize the ISS for more scientific research has brought forth opportunities for microgravity crystallization once again. A detailed description of crystallization devices designed for microgravity crystal growth can be found in DeLucas and McPherson’s review^17^. Many devices for microgravity crystallization have been developed and are available for commercial use; however, the majority are rapid-equilibration setups developed for growth of high-quality crystals for X-ray diffraction where crystal size is not a concern due to advances in beamlines, detectors, and access to high-intensity synchrotron radiation. Growing large crystals necessary for neutron diffraction require large drop volumes and can benefit from slow equilibration times. One such apparatus, the Granada Crystallization Box, is suitable for growing large crystals for neutron diffraction but is no longer commercially available^22^.

### Design of the Toledo Crystallization Box (TCB)

The GCB capillary diffusion apparatus used a gel-barrier to slow equilibration of salts used to evoke supersaturation and crystal growth^22^. The performance of the GCBs in microgravity proved highly successful for growing neutron diffraction-quality crystals. With GCBs no longer available, the TCB was developed to perform capillary diffusion crystallization in microgravity while satisfying the requirements for large crystal growth. For TS crystallization, the precipitating agent is polyethylene glycol (PEG), which has difficulty in permeating the gel barrier on the GCB. For the TCB, the thicker agarose barrier is exchanged for a thin dialysis membrane for more favorable use of PEG as a result of the much shorter pathlength of diffusion. Even using a dialysis membrane with smaller molecular weight cutoff (MWCO) than the PEG (3.5–5 kDa and 8 kDa, respectively) the PEG can still permeate the dialysis membrane due to its helical structure in solution^39^.

**Fig. 2:**
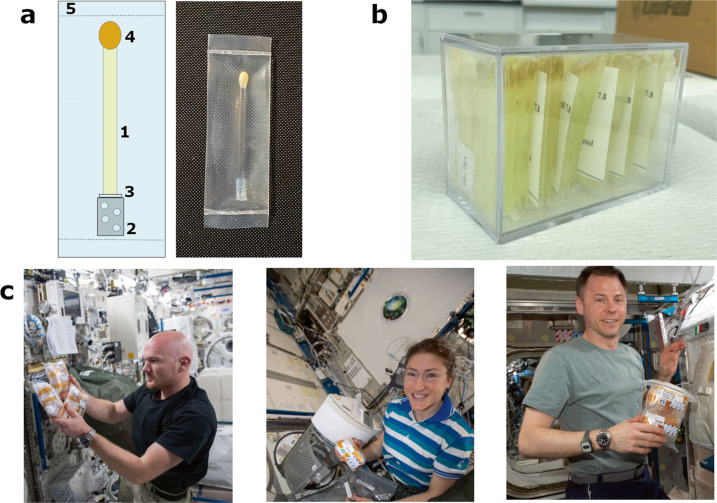
The TCB setups and flight handling. **a** Schematic of TCB capillary (left) with components indicated: (1) 2 mm inner diameter quartz capillary, 5 cm in length, filled with protein solution; (2) perforated Tygon tube cut to 1 cm length; (3) a 5 mm circular 3–5 kDa MWCO dialysis membrane fit over the end of the capillary; (4) beeswax seal; (5) heat-sealed polypropylene bag containing mother liquor. A photo of an assembled TCB capillary is shown (right). **b** A filled TCB containing 50 individual crystallization experiments. The boxes are sealed in liquid containment bags, wrapped in bubble wrap using mylar tape. **c** ESA Astronaut Alexander Gerst (left), NASA Astronauts Christina Koch (middle) and Nicklaus Hague (right) transferring sealed TCB devices into ambient storage lockers on the ISS. Photo credit: NASA.

The first TCB containers held 45 individual experiments, the second flight setup easily held 50 experiments with room for several more. The ability to conduct large scale screening in a small footprint allows for significant variation of crystallization conditions allowing broad range testing in microgravity. Each individual experiment is composed of a 5 cm long quartz capillary with a 2 mm inner diameter sealed at one end with a 3.5–5 kDa MWCO dialysis membrane secured with a short piece of Tygon tube. Holes 1 mm in diameter were punched in the tubing with a fabric hole punch (purchased at a craft supply store) to improve solvent access to the end of the capillary (Fig. 2a). The protein solution was introduced using a disposable 1 mL syringe with a blunt-end needle. Air bubbles at the dialysis membrane interface were eliminated by aspiration and the filled capillary sealed with liquified beeswax. The capillary, which holds 150 μL of protein, was positioned vertically in a small polypropylene bag containing 3 mL of mother liquor. The bags are heat sealed and placed vertically in the carrier box.

The vertical orientation in unit gravity has proven to be a critical aspect of the equilibration rates. A full study of the equilibration rates with computational modeling is presented elsewhere (CAS, TCM, V.N.D. to be published). Briefly, when the capillary is placed horizontally, the denser solution entering through the membrane flows along the side of the capillary creating eddy mixing and equilibration occurs within a few days. When the capillary is vertical, normal diffusion occurs, which is dramatically slower requiring several weeks to months to reach concentrations that promote nucleation along the entire capillary. During launch preparation, the TCBs are placed vertical in an ambient storage unit and remain vertical while the rocket is transported to the pad. The capillaries are temporarily horizontal when the rocket is raised for launch, initiating rapid equilibration, but the few hours in this position has not caused any noticeable issues.

The TCB device utilizes commercial off-the-shelf (COTS) disposable parts, is more economical than other microgravity crystallization apparatuses, and the design of the TCBs allows for variability of protein sample and crystallization conditions within the same box. Equilibration rates can be varied using different MWCO dialysis membrane and different size PEG (C.A.S., T.C.M., V.N.D. to be published). If slower equilibration is required, a small agarose gel plug can be added into the Tygon tube. With a critical feature of the TCB setup being in situ crystal growth, the quartz capillaries are the same as those used to collect neutron diffraction data. This significantly reduces risk of damage to the crystal by excessive manipulation. The TCB capillary can be disassembled, and excess liquid can be removed using a blunt-end syringe and a paper wick. Vapor H/D exchange can be performed by adding deuterated mother liquid plugs at both ends of the capillary and resealing with wax.

### Protein Crystal Growth-8 (CASIS PCG-8) SpaceX CRS-15 Mission test experiments

The first flight of the TCB device was assigned experiment number PCG-8 by CASIS and was in the manifest aboard SpaceX CRS-15 on June 29, 2018, docking at the ISS on July 2, 2018. The TCB devices returned to Earth on August 3, 2018, spending 31 days aboard the ISS in an ambient storage locker, which was set to 22 °C (Fig. 2c). The Fisher Scientific Low Temperature Incubator (Model # FFU20F9FW0) used for ground control experiments was also set to 22 °C to maintain consistency. Upon visual inspection of the capillaries returned from PCG-8 and corresponding ground controls, the flight crystals appeared to be single with fewer flaws. Both ground and flight capillaries contained an abundance of crystals, with the microgravity crystals exhibiting more of a three-dimensional shape compared to the thin rods in the ground control setups. Room temperature X-ray diffraction data were collected at the IMCA-CAT 17-ID-B microfocus beamline on multiple H-TS crystals to compare PCG-8 and ground controls. X-ray damage severely limited our analysis as several passes of repositioned crystals were merged to obtain complete data sets. Subjectively, processed data from comparable-sized crystals showed that microgravity-grown crystals diffracted to higher resolutions (typically ~2 Å resolution for microgravity verses >3 Å resolution for ground controls) and had lower mosaicity values (0.19° for microgravity verses 0.27° for ground controls). These quality checks provided enough evidence to support an additional longer duration ISS experiment. Follow-up visual assessment 2 months after the return of the TCBs showed new growth and nucleation points. At that time, preliminary equilibration data on the TCB capillaries in the vertical position with the 10–12 kDa MWCO dialysis membrane indicated the capillaries were not fully equilibrated after 13 days. Flight setups were prepared with smaller 3.5–5 kDa MWCO membranes, which should take longer to equilibrate. This data combined with the observation of new growth and nucleation, suggested the TCB capillaries did not reach equilibration aboard the ISS on PCG-8. Longer flight duration was requested for the next flight to allow for complete equilibration and growth.

### Production experiments on CASIS PCG-15 SpaceX CRS-18 Mission

**Fig. 3:**
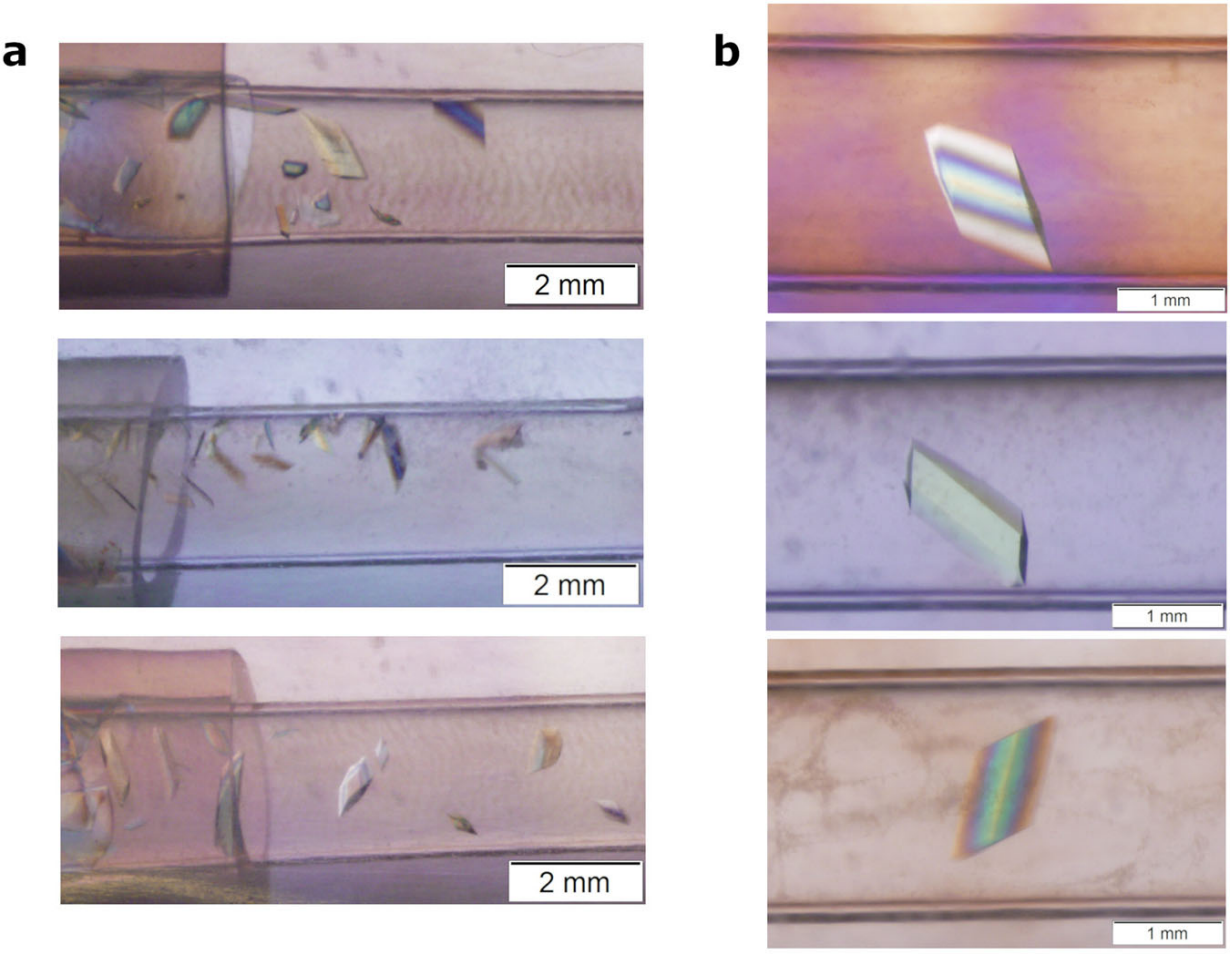
Perdeuterated TS crystals from micro- and unit gravity PCG-15 experiments. The crystals were visually assessed and imaged at Oak Ridge National Laboratory following their retrieval from Johnson Space Center after CRS-18 capsule splashdown. **a** Representative pictures of larger ground control pD-TS crystals grown in TCB capillaries with 2 mm scale bar. The crystals grew near the bottom of the capillary and were relatively small with some inclusion defects. **b** Representative pictures of larger microgravity-grown pD-TS crystals from TCB capillaries with 1 mm scale bar. Flight crystals were single and large, nearly spanning the width of the capillary (2 mm).

The second flight of TCBs was assigned experiment number CASIS PCG-15 and was aboard SpaceX CRS-18 on July 25, 2019, docking on the ISS on July 27, 2019. The TCB devices returned with SpaceX CRS-19 on January 7, 2020, spending about 6 months aboard the ISS. Again, both flight and ground controls were stored at ambient 22 °C. The PCG-15 experiments included pD-TS in addition to H-TS samples. Examination of pD-TS crystals from PCG-15 and ground controls revealed the microgravity had improved the volume of the crystals by ~5-fold (Fig. 3).

The microgravity-grown pD-TS crystals were visually free of flaws or damage, while inclusions, multinucleation, and other flaws could be seen in most of the ground control crystals. In addition, the TCB capillaries from flight had a greater propensity for growing near the middle, whereas ground control crystals tended to grow closer to the bottom of the capillaries. There was no distinguishable trend in which conditions produced the largest crystals, with large crystals appearing at all sampled percentages of PEG in the microgravity TCBs. We also discovered that adaption from vapor diffusion to optimization of crystallization in the TCB capillaries produced better crystals in both microgravity and unit gravity (Table 1).

**Table 1:** Preliminary neutron and X-ray data collection statistics.

	pD-TS neutron microgravity	pD-TS X-ray microgravity	pD-TS X-ray ground control
Beamline	LADI-III, ILL	Rigaku HighFlux HomeLab, ORNL	Rigaku HighFlux HomeLab, ORNL
Temperature (K)	293	293	293
Wavelength (Å)	2.85–3.80	1.54	1.54
Exposure time	24 h	5 s	5 s
No. of images	57	200	170
Resolution (Å)	52.36–2.10 (2.21–2.10)	91.91–1.80 (1.86–1.80)	91.99–1.90 (1.97–1.90)
Space group	C 1 2 1	C 1 2 1	C 1 2 1
Unit cell *a, b, c* (Å)	184.51, 61.87, 67.68	184.51, 61.87, 67.68	184.62, 62.07, 67.67
Unit cell *α*, *β*, *γ* (°)	90.00, 94.74, 90.00	90.00, 94.74, 90.00	90, 94.73, 90
Unique reflections	30,993 (3386)	65,668 (5693)	57,476 (5380)
Multiplicity	6.8 (5.7)	3.0 (2.0)	2.4 (1.7)
Completeness (%)	70.1 (52.5)	93.0 (80.6)	95.2 (89.1)
*R*_merge_	0.182 (0.410)	0.052 (0.311)	0.047 (0.292)
*R*_pim_	0.065 (0.138)	0.033 (0.283)	0.034 (0.291)
*I*/ *σ*(*I*)	10.4 (2.0)	26.59 (2.39)	19.65 (2.41)

Statistics for the highest-resolution shell are shown in parentheses.

As the average room-temperature X-ray diffraction resolution for flight and ground control pD-TS crystals was similar, visually equivalent crystals were chosen for neutron diffraction comparison on IMAGINE. Selection was limited by the size of the crystals from ground control experiments, with the largest unit gravity crystal reaching 0.2 mm^3^. Comparable crystals from microgravity and unit gravity with 0.2 mm^3^ volume were vapor H/D exchanged in situ against their crystallization solution prepared in D_2_O (50 mM Bicine pH 7.8, 1 mM EDTA, 0.2 mM PLP, 2 mM spermine, and 8% PEG 8000) for a minimum of 2 weeks prior to data collection.

**Fig. 4:**
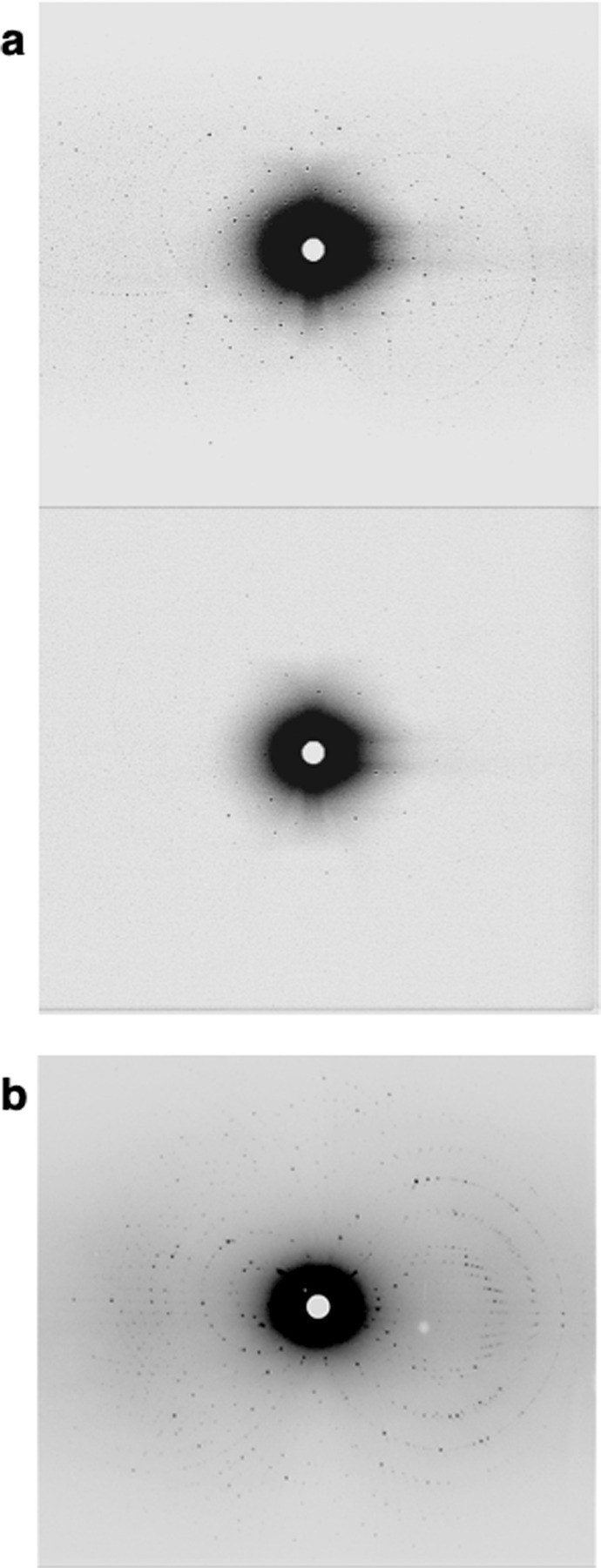
Comparison of neutron diffraction of perdeuterated TS crystals from PCG-15. **a** 16-h Laue diffraction image collected using wavelengths 2.8 to 10 Å from 0.2 mm^3^ microgravity-grown pD-TS crystal (top). The flight crystal diffracted to 3.2 Å. 16-h Laue diffraction image (2.8–10 Å) from 0.2 mm^3^ ground control pD-TS crystal (bottom). The ground control crystal diffracted to 4.0 Å and exhibited a much weaker diffraction pattern. Neutron diffraction data was collected on IMAGINE at ORNL. **b** A quasi-Laue diffraction image (2.85–3.8 Å) of a large (>1 mm^3^) microgravity-grown pD-TS crystal 19-h collected on LADI-III at ILL, which diffracted to 2.1 Å resolution. A 5-fold increase in crystal volume significantly improved diffraction resolution.

A 16-h exposure Laue image and 24-h exposure quasi-Laue image were captured for both crystals. Diffraction spots could be indexed and integrated for both crystals. The microgravity-grown crystal diffracted neutrons to 3.2 Å and exhibited stronger diffraction spots than the ground control crystal, which only diffracted neutrons to 4.0 Å (Fig. 4a). PCG-15 pD-TS flight crystals were sent to the Institut Laue-Langevin (ILL) in Grenoble, France. Data were collected on a >1 mm^3^ pD-TS crystal from microgravity on beamline LADI-III, yielding diffraction resolution to 2.1 Å (Fig. 4b). A total of 57 quasi-Laue images were collected over 4 orientations of the crystal. The first 44 images were collected during ILL High Flux Reactor cycle 190, which ran at reduced power, 42 MW. The remaining 13 images were collected during cycle 191 when the reactor was at full power, 54 MW. Neutron data collection statistics are shown in Table 1. It should be noted that IMAGINE and LADI-III are instruments of comparable design and the significant improvement in resolution can be attributed to the larger volume of the crystal used in data collection and the brighter neutron beam at the LADI-III beamline.

Unlike X-ray diffraction, neutron diffraction is a non-destructive technique and can directly detect hydrogen or deuteron positions in biomacromolecules even at moderate resolutions^12^. While providing more complete information about all atoms in the protein, the low flux of neutron sources and difficulty in data collection yield data sets with limited completeness, typically 65– 80% coverage and lower resolution compared to X-ray data. Joint refinement using both X-ray and neutron data simultaneously is a procedure that overcomes the data quality issues of neutron diffraction^40^. For joint refinement to work, the crystal data sets need to be isomorphous. X-ray data from both ground and flight crystals of perdeuterated TS (Table 1) are amenable to use in our planned joint refinement. Determination of hydrogen positions is important in understanding enzyme catalysis and can offer information about substrate binding and protein dynamics, as well^41^. In addition, neutron structures have been shown to be adept at providing accurate input models for quantum chemical calculations for understanding catalysis on the electronic level^42,43^. Neutron diffraction is a critical technique in studying PLP-dependent enzymes as it allows us to capture static images of the protein active site protonation states at different steps in the reaction mechanism. These snapshots can be pieced together to reveal the how protonation of the PLP cofactor influences catalysis.

In conclusion, we have designed the TCB device to facilitate slow equilibration capillary-dialysis crystallization for the production of large, single crystals for neutron diffraction. The design was tested during the CASIS PCG-8 experiment where it demonstrated improved diffraction resolution and crystal packing of H-TS. Longer duration experiments during SpaceX CRS-18 through CRS-19 (CASIS PCG-15) produced several crystals ≥1 mm^3^ of pD-TS. Neutron diffraction comparison of flight crystals and ground controls on IMAGINE at ORNL confirmed microgravity improved the crystal’s ability to diffract neutrons. Microgravity-grown pD-TS crystals were sent to ILL and diffracted to 2.1 Å on LADI-III. The success of microgravity crystal growth with the TCB device for perdeuterated tryptophan synthase solved a major hinderance of neutron crystallography, providing a methodology for future crystal growth.

## Data Availability

The data that support the findings of this study are available upon reasonable request from the corresponding author [T.C.M.]. The data are not yet publicly available. The data will be released to the Protein Data Bank upon completion of the crystal structures, which are to be published in a subsequent manuscript.
